# Supplementary material for: Validation of an Automated Scoring Algorithm That Assesses Eye Exploration in a 3-Dimensional Virtual Reality Environment Using Eye-Tracking Sensors
Source: Sensors (Basel). 2025 May 26;25(11):3331. doi: 10.3390/s25113331 (PMC12158043; doi:10.3390/s25113331)

Supplementary material for:
Validation of an Automated Scoring Algorithm that Assesses Eye Exploration in 3-Dimensional Virtual Reality Environment Using Eye Tracking Sensors

A. List of recent eye tracking related gaze detection algorithms:

1. ADGaze: Anisotropic Gaussian Label Distribution Learning for fine-grained gaze estimation;

[Li, D., Wang, S., Zhao, W., Kang, L., Dong, L., Wang, J., & Wang, X. (2025). ADGaze: Anisotropic Gaussian Label Distribution Learning for fine-grained gaze estimation. Pattern Recognition, 164, 111536.]

2. DADL: Double Asymmetric Distribution Learning for head pose estimation in wisdom museum;

[Zhao, W., Wang, S., Wang, X., Li, D., Wang, J., Lai, C., & Li, X. (2024). Dadl: Double asymmetric distribution learning for head pose estimation in wisdom museum. Journal of King Saud University-Computer and Information Sciences, 36(1), 101869.]

3. GCANet: Geometry cues-aware facial expression recognition based on graph convolutional networks;

[Wang, S., Zhao, A., Lai, C., Zhang, Q., Li, D., Gao, Y., ... & Wang, X. (2023). Gcanet: Geometry cues-aware facial expression recognition based on graph convolutional networks. Journal of King Saud University-Computer and Information Sciences, 35(7), 101605.]

4. MFDNet: Collaborative Poses Perception and Matrix Fisher Distribution for Head Pose Estimation;

[Liu, H., Fang, S., Zhang, Z., Li, D., Lin, K., & Wang, J. (2021). MFDNet: Collaborative poses perception and matrix Fisher distribution for head pose estimation. IEEE Transactions on Multimedia, 24, 2449-2460.]

5. Precise head pose estimation on HPD5A database for attention recognition based on convolutional

Neural network in human-computer interaction;

[Liu, H., Li, D., Wang, X., Liu, L., Zhang, Z., & Subramanian, S. (2021). Precise head pose estimation on HPD5A database for attention recognition based on convolutional neural network in human-computer interaction. Infrared Physics & Technology, 116, 103740.]

Supplementary material for:
Validation of an Automated Scoring Algorithm that Assesses Eye Exploration in 3-Dimensional Virtual Reality Environment Using Eye Tracking Sensors

Supporting material for the *Methods* section- Subsection: *Participants*

Table S1: Inclusions and exclusion criteria

**Inclusion criteria**	**Exclusion criteria**
Cognitively normal or people with AD aged 60-90	Neck problems including chronic or acute pain, past or present vertebral fractures
Patients with AD will meet NIA-AA core clinical criteria for probable AD dementia	Severe dementia- MMSE<12
Absence of significant dementia-related emotional/behavioral symptoms other than apathy	Behavioral problems that preclude compliance with study protocol;
Cognitive performance will be judged by the treating physician as such that enables compliance with study protocol instructions	Vestibular problems
Participants should be ambulatory, able to walk independently for an hour (with pauses) on the treadmill in the VR devise	Existence of other active medical diagnoses that may compromise participants' ability to walk on the treadmill (e.g. unstable cardiac or pulmonary disease).
For patients with dementia and apathy: NPI-apathy subscale score ≥ 4.	
Clinical assessment by a geriatric psychiatrist and a neuropsychologist and complete cognitive, affective, and behavioral scales of the study population by the following completed forms:<br>• Mini Mental State Examination<br>• Montreal cognitive assessment battery<br>• Frontal assessment battery<br>• Lille apathy rating scale<br>• Cornell scale for depression in dementia<br>• Geriatric Depression Scale - 15 item version + caregiver version	

Supplementary material for:
Validation of an Automated Scoring Algorithm that Assesses Eye Exploration in 3-Dimensional Virtual Reality Environment Using Eye Tracking Sensors

B. Supporting material for the *Methods* section- Subsection: *Apathy study - procedure*

Explanations on questioners and evaluations used in this study

MOCA (Montreal Cognitive Assessment: This dementia test can produce pathological results in numerous neurological and psychiatric disorders, including Alzheimer's disease, vascular dementia, Levy body dementia, frontotemporal dementia, Parkinson's disease, Huntington's disease, stroke, multiple sclerosis, ALS, endogenous depression, schizophrenia, sleep apnea, drug abuse, brain tumors, traumatic brain injury, cerebral involvement in HIV and other systemic infections. Therefore, MOCA is no disease-specific test. It consists of 30 questions, each of which can be awarded one point, resulting in a maximum of 30 points. A total score below 26 points indicates a pathological result in the sense of cognitive impairment.

LARS (Lille Apathy Rating Scale): This test consists of 33 items covering nine different domains. The score is based on a dichotomous scale from -36 to +36. The developers of the test empirically determined a cutoff of -16. Values from -36 to -16 are declared as normal, while values greater than -16 can be regarded as pathological; the higher the numerical value, the more pronounced the apathy. In later applications, a finer gradation was determined and proposed:
-36 to -21: normal, no apathy;
-20 to -16: mild apathy;
-15 to -9: moderate apathy;
> -9: severe apathy.

FAB (Frontal Assessment Battery): This test specifically assesses forms of frontotemporal dementia and can be helpful in differentiating it from Alzheimer's dementia. To assess executive functioning, a total of six categories are used focused on executive functions. A maximum of three points can be awarded per category (normal finding), low graded with 2, 1, or 0 points depending on the severity of dementia. The maximum achievable value (ideal normal score) is 18 points; the theoretically worst score would be zero points. Twelve points are considered the cutoff. Thus, anyone scoring more than twelve points is not considered to have dementia; lower total scores indicate dementia, and the lower the numerical result, the more severe it is.

GDS (Geriatric Depression Scale): This survey instrument consists of 15 individual questions, each of which can be answered with yes or no. If answered affirmatively, this indicates depressive symptoms. The range of measured values thus goes from zero (ideally normal, no depression) to a maximum of 15 (most severe depression). The following gradual classification has been established:
0-5 points: normal findings,
5-10 points: mild to moderate depression,
10-15 points: severe depression.

Supplementary material for:
Validation of an Automated Scoring Algorithm that Assesses Eye Exploration in 3-Dimensional Virtual Reality Environment Using Eye Tracking Sensors

C. Demonstration of the VR scene with eye movement

Link to video file:
https://drive.google.com/file/d/1GnKlxx7jArTuGC72ncKGS8pTL943Uf5Y/view?usp=sharing

D. Supporting material for the *Methods* section- Subsection: *Algorithm and statistical analysis*

Figure S-1: Detailed flow chart of the scoring algorithm **(see next page)**

Supplementary material for:
Validation of an Automated Scoring Algorithm that Assesses Eye Exploration in 3-Dimensional Virtual Reality Environment Using Eye Tracking Sensors

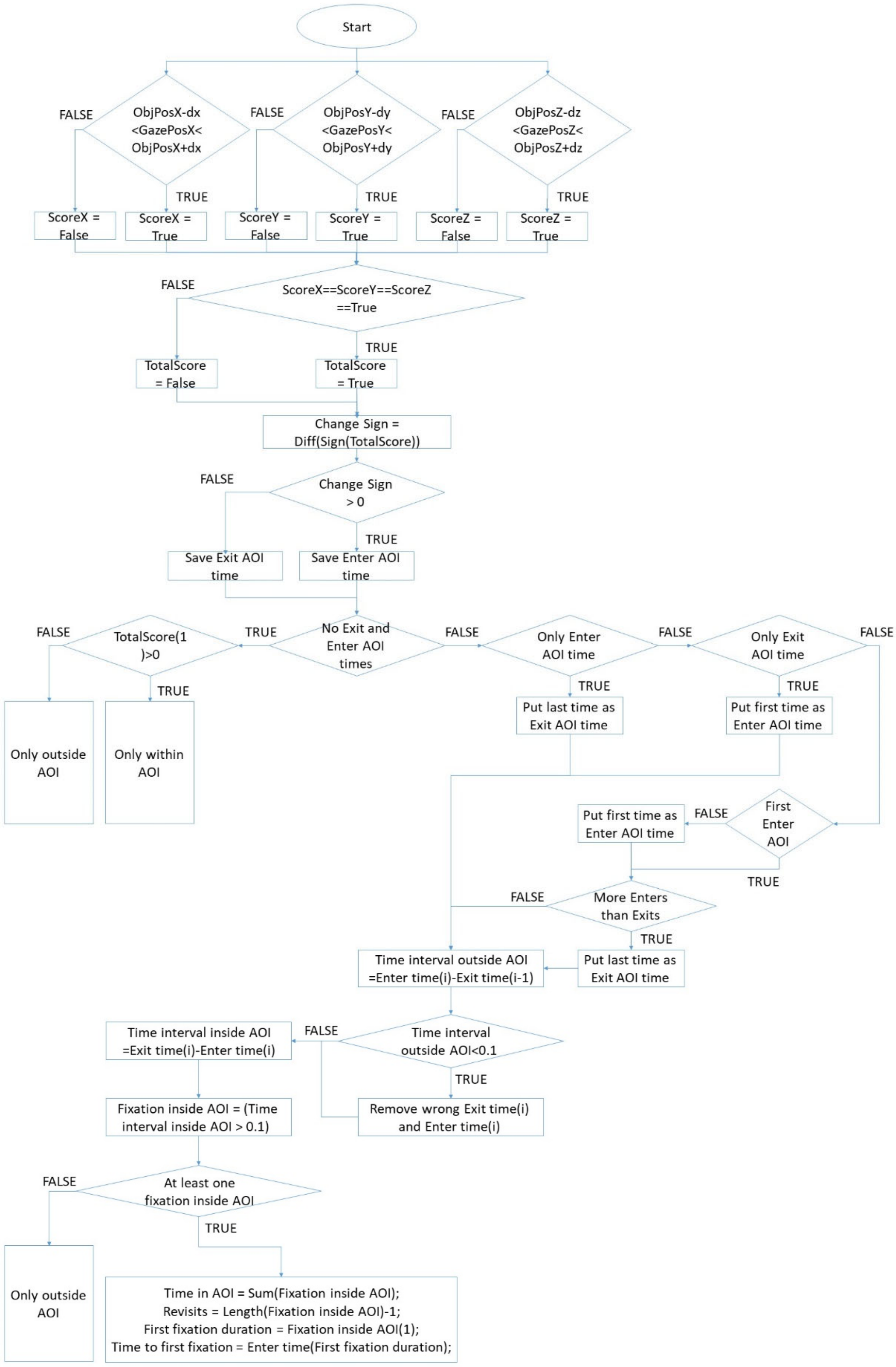

Supplement: Supplementary file 1 [file sensors-25-03331-s001.zip › Supplementary materials - Revised version.pdf]
