# Supplementary figures and images for: Validation of an Automated Scoring Algorithm That Assesses Eye Exploration in a 3-Dimensional Virtual Reality Environment Using Eye-Tracking Sensors
Source: Sensors (Basel). 2025 May 26;25(11):3331. doi: 10.3390/s25113331 (PMC12158043; doi:10.3390/s25113331)

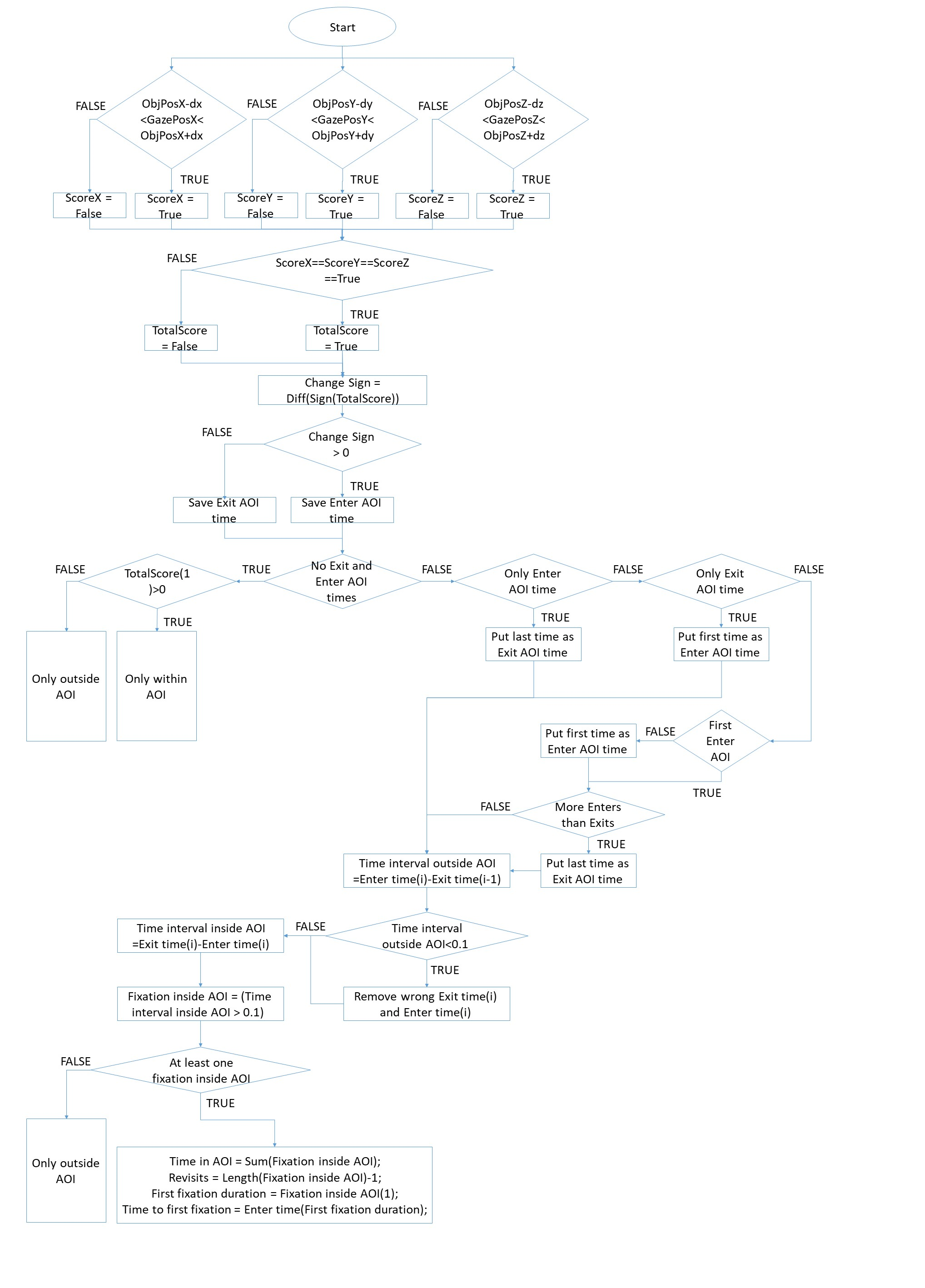

Supplement: Supplementary file 1 [file sensors-25-03331-s001.zip › Figure S-1.tiff]
